# The Shurly-Gibbes Treatment of Tuberculosis

**Published:** 1892-12

**Authors:** H. Longstreet Taylor

**Affiliations:** Asheville, N. C.


### THE SHURLY-GIBBES TREATMENT OF TUBERCULOSIS.*

By H. LONGSTREET TAYLOR, M. D., A. M.,
Asheville, N. C.

After briefly going over the history of tuberculin and expressing a conviction that good was certain to come from this discovery of Koch's, Dr. Taylor went on to say that the creosote treatment was now probably the most popular with the profession. While he was ready to admit that it was not without its good, yet it does not go on to a cure, and the chief reason is that it sooner or later upsets the stomach—a most important organ to the consumptive—who has need of all the nourishment he can possibly obtain. Consequently he now uses in properly selected cases the Shurly-Gibbes injections of iodine and the chloride of gold and sodium, with inhalations of chlorine gas or of a spray containing a strongly disinfecting solution.

A history of this treatment was given, with reasons why the profession has been so slow to adopt it, and a brief *résumé* of the cases that have been reported in literature, now amounting to several hundred, which show a large percentage of cases, many freed from the presence of tubercle bacilli and a great diminution in the number of bacilli in others. That these remedies would cause fever to disappear, night sweats to cease, expectoration to grow less and lose its load of tubercle bacilli, sleep improve, weight to increase and general nutrition to be built up. When medicine has done this much, then a change of surroundings, mode of life, and when possible climate, together with constant medical supervision and management, we may expect to see many cases get well. Then followed a short sketch of method of management at Asheville and a plea for the profession to keep far advanced patients at home. After a few case histories, in conclusion he said, I have now used the

*Abstract of paper read before the Mississippi Valley Association, October, 1892.

Shurly-Gibbes treatment for eighteen months on upwards of sixty cases, with the general management detailed above and am very well satisfied with the results. Some of the most encouraging of these to me were hopeless and eventually fatal cases, which by the improvement in their condition and the prolongation of their lives, showed beyond a doubt that these remedies can check the advance of the disease.

The discussion was then taken part in by Dr. Eichberg, Dr. Thrasher, and Dr. Thompson, and closed by Dr. Taylor.

In closing the discussion Dr. Longstreet Taylor insisted that the cases reported showed that the improvement was not alone due to suggestion or to Asheville climate, as these had both been tried and had failed, and that improvement only became permanent after a thorough course of remedies. And that he used a modification of the trachial injections of creosote in the form of an albolene spray, carrying 10 per cent. of creosote and 3 per cent. of menthol, which deeply inhaled and retained, seemed asepsis of the bronchial tubes and gave the patients marked relief from paroxysms of coughing.

